# Applying Adult Learning Best Practices to Design Immunization Training for Health Care Workers in Ghana

**DOI:** 10.9745/GHSP-D-21-00090

**Published:** 2021-09-30

**Authors:** Denise Traicoff, Dieula Delissaint Tchoualeu, Joseph Opare, Melissa Wardle, Pamela Quaye, Hardeep S. Sandhu, George Bonsu

**Affiliations:** a Centers for Disease Control and Prevention Global Immunization Division, Atlanta, GA, USA.; b African Field Epidemiology Network, Accra, Ghana.; c Ghana Health Service, Accra, Ghana.

## Abstract

**Introduction:**

A 2016 assessment of frontline health care workers (HCWs) in Ghana identified knowledge, skill, and attitude gaps related to immunization during the second year of life (2YL). The U.S. Centers for Disease Control and Prevention subsequently supported the Ghana Health Service Immunization Program to apply best practices of adult learning and training of trainers (TOT) for a cascade training program for 2YL.

**Methods:**

Five districts from each of the 3 regions (Greater Accra, Northern, and Volta) were selected for the TOT based on key measles and rubella vaccination coverage indicators. The design incorporated best practices of adult learning and TOT. The curriculum integrated 3 major topical themes: technical (immunization topics), operational, and training adults. The technical and operational content was based on HCW tasks most directly affecting 2YL objectives. A cross-functional team developed all classroom, field activity, and training evaluation materials.

**Results:**

Seventy-four participants attended TOT workshops in 2017. Based on a rubric defined by the course designers, 99% of the participants reported an acceptable level of confidence to apply and teach the course content. After the TOTs, participants conducted 65 workshops, 43 field visits, and 4 review meetings, reaching 1,378 HCWs within 7 months. Fifty-four percent of HCWs who received training from TOT participants reported an acceptable level of confidence in using the skills, and 92% reported they would prioritize applying the skills acquired during the training.

**Discussion:**

The success factors for effective adult learning and TOT can be applied to design and implement high-quality TOT even in resource-limited settings. The factors include using a variety of approaches, spending enough class time to prepare TOT participants for their training role, setting specific expectations for cascading the training, and following up through mentorship and reporting. Strong collaboration across the administrative levels of the Ghana Health Service enabled cascade training.

## Abstract (precis)

Best practices of adult learning were used to develop a training of trainers program for the Ghana Health Service immunization workforce. The program supported translating learning to behavior change, used class time for practice-teaching and action plan development, linked formal instruction with specific activities, and offered follow-up mentorship.

## Teaser

**Key Findings:**

- The published literature regarding adult learning and training of trainers provides practical guidance for developing performance-based training of trainers, such as for the Ghana Health Service immunization workforce.
- Training of trainers (TOT), while a common capacity-building strategy, presents several risks that must be carefully mitigated.
- The Ghana 2YL TOT was designed with a systems approach, including audience selection, class activities, evaluation methods, and postworkshop action planning.
- Performance-focused class activities, including practice teaching, increased participants’ confidence as technicians and trainers.

**Key Implications:**

- Program managers should consider the risks involved in TOTs and be prepared to support the cascade with human and financial resources.
- Technical experts can take practical steps to ensure TOTs follow adult learning and TOT best practices.
- TOT designers should include opportunities for practice teaching and feedback to prepare participants for their training role.
- Program managers and designers should develop reporting tools and processes and monitor trainers’ field activities.

## INTRODUCTION

Since 2006, the Ghana Health Service Expanded Programme on Immunization (GEPI) has sustained coverage levels of at least 85% with the first dose of the measles-containing vaccine (MCV1).^1^ In 2012, Ghana introduced the second dose of MCV (MCV2), yet coverage remained well below the intended target, reaching only 63% in 2015.^2^ This level is well below the recommended 95% target needed to stop transmission of measles. Additionally, there were gaps in immunization coverage among districts for other vaccines, with 1 in 5 districts not achieving the goal of >80% coverage with pentavalent vaccine in 2015.^2^

To strengthen country capacity to prevent, detect, and respond to vaccine-preventable disease threats, GEPI, in collaboration with the United States Centers for Disease Control and Prevention (CDC), led an initiative to strengthen the demand and delivery of vaccines, organized under the term “second year of life” (2YL). Three of the 10 administrative regions, Greater Accra, Northern, and Volta, were selected for the interventions because they contained the greatest number of districts with low MCV2 coverage and inequitable access to immunization services.^3^

To understand the underlying causes of performance gaps contributing to low MCV2 coverage, GEPI and CDC surveyed the knowledge, beliefs, and practices of caregivers and health care workers (HCWs) in 2016, and reviewed the availability of resources at the health facility level that support the 18-month routine immunization visit.^3^ This baseline assessment identified knowledge, skill, and attitude gaps among HCWs in crucial aspects of immunization service delivery for children in their 2YL, particularly concerning national immunization policies, data recording and reporting, tracking children who did not return for additional vaccinations they were due to receive (known as defaulter tracking), and communicating with caregivers. These findings informed the design, implementation, and evaluation of an array of interventions that CDC supported, such as organizing data improvement teams, conducting social mobilization campaigns, and training staff at the district and health facility level. One training intervention was a 2.5-day pilot workshop for community health nurses and their supervisors in Greater Accra. After positive results from the pilot, GEPI requested CDC assistance in scaling up the training using a training of trainers (TOT) approach.

A baseline assessment identified knowledge, skill, and attitude gaps among HCWs in crucial aspects of immunization service delivery for children in their second year of life.

Also known as cascade training, TOT is an internationally recognized method for training a large cohort of learners who are usually dispersed geographically.^4^ TOTs rely on a small group of qualified (“master”) trainers who train a larger group of individuals, who then in turn train others. This process continues until the entire learner population has been reached. TOTs have been shown to be effective in health settings.^5^ In low-resource countries, TOTs are generally conducted entirely through traditional face-to-face classroom instruction, as technology constraints limit distance-based modalities. Although a popular method in both the private sector and public institutions, the TOT model faces several challenges that dilute its effectiveness. For example, studies have indicated several drawbacks to using experts as trainers. Frequently, these individuals are selected based on their technical expertise, a characteristic that does not necessarily translate into an ability to transfer knowledge to novices.^6^^,^^7^

The training of trainers model faces several challenges that dilute its effectiveness.

Hahn et al.^8^ and Orfaly et al.^9^ point out additional concerns including fidelity to the content, both in terms of accuracy and in adjusting the emphases of content based on the specific target audience. They also describe a more common challenge centered on follow-through, which is the number of trainers who go on to conduct trainings of their own. Pearce et al.^5^ cite turnover of trainers and trainees as threats to long-term impact. In addition, like other workplace learning interventions, factors such as motivation, supervisor support, and triggered action planning can affect whether the trainers apply their new skills.^10^ Finally, it is often challenging to design and implement a valid evaluation method. A key question is whether TOT success should be measured based on the number of trainers trained, the trainers’ self-assessment of confidence as a trainer, or the improved proficiency or behaviors by the learners at the lowest level of the training chain. Still, as Hayes^11^ points out, it may not be the TOT model itself that is the problem, but the way it is implemented. Hayes^11^ has proposed 5 critical success factors for TOT in the educational setting that hold promise as a best practice in the public health environment:

1. The method of conducting the training must be experiential and reflective rather than transmissive (i.e., one-directional, such as lectures).
2. The training content and delivery method must be open to reinterpretation rather than prescribe a rigid adherence to a predefined method (i.e., should be sensitive to the local context, while maintaining fidelity to the intent of the content).
3. Expertise must be diffused through the system as widely as possible.
4. Decentralization of responsibilities within the cascade structure is desirable.
5. A cross-section of stakeholders must be involved in the preparation of training materials.

These practices harmonize and overlap with the body of knowledge related to adult learning, as described by andragogy scholars such as Knowles et al.^12^ and Dirksen.^13^ Specifically, Knowles et al.^12^ proposed 4 principles to apply to adult learning:

1. Adults want to know why they need to learn something.
2. Adults need to learn experientially, based on tasks not theories.
3. Adults approach learning as problem solving or to accomplish a specific goal.
4. Adults learn best when the topic is of immediate value.

This article describes our experience incorporating adult learning best practices and the recommendations of Hayes^11^ into the design and delivery of the TOT intervention for Ghana 2YL and proposes their application in public health workforce development.

## METHODS

The 2 objectives of the TOT were to improve the competency of district health management teams (DHMTs) regarding primary 2YL vaccination services and to improve their capacity as trainers. The training interventions were implemented in all 3 2YL regions: Greater Accra, Northern, and Volta.^3^ The target audience included staff at the regional and district levels who were already responsible for providing supportive supervision at the immediate lower level. Five districts from each region were purposively selected to include 2 high-performing districts and 3 low-performing districts based on their MCV2 vaccine coverage, drop-out rates between MCV1 and MCV2, and other Expanded Programme on Immunization (EPI) performance indicators. In each selected district, participants included the regional health management team (RHMT) and approximately 4 DHMT members. Participants worked as a group with their district throughout the workshop.

The TOT was intended to improve competency of DHMTs regarding primary 2YL vaccination services and to improve their capacity as trainers.

### TOT Curriculum Design

To achieve the workshop objectives, the curriculum was designed to integrate 3 major themes: technical, operational, and training adults. Training materials were developed by a cross-functional team composed of GHS and CDC staff with expertise in national immunization policy, immunization practices, and adult learning. The technical and operational content was based on the knowledge, skill, and attitude gaps identified by the baseline assessment and is summarized in Table 1.

**TABLE 1.** Ghana Training of Trainer Workshop Topics To Improve Competency in Primary Second Year of Life Vaccination Services

Technical Topics	Operational Topics	Adult Learning
Regional EPI performanceEPI policies2YL projectMeasles immunogenicityMonitoring data for actionMonthly reportingData analysisDefaulter trackingCommunicating with caregivers	Problem analysis and prioritizationProcess analysis and improvementBest practices of supportive supervision	Characteristics of adult learnersFive moments of learning needClassroom delivery techniquesManaging the classroomChoosing the best learning strategy: classroom, small groups, one-on-oneDeveloping training action plans

Abbreviations: 2YL, second year of life; EPI, Expanded Programme on Immunization; TOT, training of trainers.

The 5-day workshop included 2.5 days of technical content, such as GEPI 2YL policy and data analysis, and operational content, such as problem analysis and interpersonal skills. The remaining workshop time was dedicated to strengthening participants’ ability and confidence to plan and deliver training to HCWs in their districts. After each workshop, the team made changes to the materials, schedule, or tools to improve and localize subsequent workshops.

### Application of TOT Best Practices

The curriculum design was guided by a fundamental principle for adult learning: workplace training should be learner focused and performance based.^14^ Thus, all technical and operational content was developed and organized based on specific immunization tasks, using participants’ own data as much as possible. Hayes’ 5 success factors^11^ were integrated into the design and used as a quality check.

The curriculum design was guided by a fundamental principle for adult learning: workplace training should be learner focused and performance based.

#### Experiential and Reflective Learning

Adhering to both andragogy principles of Knowles et al.^12^ and recommendations of Hayes^11^, the sessions were highly interactive and used the participants’ local context and local immunization data. The workshop introduced practical aspects of adult learning to help the trainers plan and deliver the 2YL training themselves. Participants were introduced to the 5 moments of learning need, an adult learning model that helps trainers determine what types of content should be taught in a formal setting and what types of content can be learned informally using job aids or coaching.^15^ This model also reinforces the importance of on-the-job coaching as a factor to encourage learning application. A master trainer conducted review sessions at the end of each day, to review not only the technical content but also the teaching methods. For example, the master trainer led discussions to help participants determine the best training delivery method: workshop, coaching during a health facility visit, or small group review meetings. These daily sessions enabled participants to reflect on what they learned and how they would diffuse the information.

#### Open to Reinterpretation

The organizers wanted to balance flexibility while promoting content fidelity. Therefore, the training materials for each topic included formal presentations with speaker notes, exercises with answer keys, and a facilitator’s guide. In-class discussions and activities helped participants consider how to localize the content. A detailed lesson plan for the TOT component of the workshop, as well as examples of participant materials, is available in a Supplement.

Teachback was a key activity to support these principles. Participants formed teams of 2 and chose a 20-minute lesson from the 2YL curriculum. They were given electronic versions of all training materials to prepare for their teachback lesson. They were encouraged to customize their lesson while adhering to the lesson’s learning objective(s). The master trainers completed a feedback worksheet as they observed the lessons. The participants also received written feedback from their peers immediately after they taught their lesson. Finally, participants applied what they had learned and their knowledge of their district to develop a preliminary training plan, mapping out the number, location, and timeframe of site visits and workshops they expected to deliver.

Participants had an immediate opportunity to practice teaching the content and to plan how they would deliver the training to others.

#### Diffusion of Expertise and Decentralization of Responsibilities

Addressing 2 of Hayes’ 5 factors,^11^ the design also included components to support the participants after the training as they implemented their plans in the 3 regions. In addition to obtaining electronic versions of all materials, they were mentored by national and regional staff. The participants were encouraged to assess knowledge and skill gaps of the HCWs in their jurisdiction. Their assessment results could help the participants deliver the appropriate content and delivery method and monitor results. Application of operational topics could come in the form of teaching others or applying what they learned to the way they do their work, such as using the lesson *Best practices of supportive supervision* to improve one’s own supervision practices. GEPI instituted a requirement for DHMTs to submit field activity reports specific to this initiative to the higher levels.

#### Cross-Section of Stakeholders

The cross-functional team that developed the content also served as master trainers and modeled adult learning best practices when leading the classroom sessions. In addition, RHMTs facilitated the classroom activities to provide local context and joined the national-level staff as mentors for the post-classroom field activities. Table 2 summarizes the program design as it relates to the key practices of effective adult learning and to Hayes’^11^ recommendations.

**TABLE 2.** Summary of Adoption of Adult Learning and Training of Trainers Best Practices

**Best Practice**	**Application for Ghana TOT**
Adult learning	
Adults want to know why they need to learn something.	Lessons on foundations of adult learning, to help participants understand the strategies behind the curriculum design Practical, performance-based content for technical, operational, and adult-learning topics
Adults need to learn experientially.	Interactive small group work Activities to apply knowledge Minimal theoretical lectures
Adults approach learning as problem solving.	Daily sessions to review content from the trainer’s point of view, such as tips for explaining confusing content Scenario-based exercises and role plays
Adults learn best when the topic is of immediate value.	Use of local examples and data Action planning: developing field training plans *Handling challenging situations* handout to support trainers when managing problem participants
TOT	
Method of training must be experiential and reflective	Interactive sessions, simulating the work environment Teachback sessions to provide the opportunity to practice teaching in a safe-fail environment and receive constructive feedback from master trainers and peers Participant individual development plans, to self-reflect and develop a plan to improve their teaching skills Multiple training events at the health facility level, which reinforced training skills while diffusing the knowledge
Training content and delivery method must be open to reinterpretation	Participant and instructor training materials provided in easily editable format, while at the same time adhering to national immunization policy standards Class discussions to identify ways to localize content Guidelines to help trainers choose best training delivery strategy
Expertise must be diffused through the system	Peer and mentor feedback during and after the workshop Participant action plans for delivery to health facility level National, regional, and district-level supervision and mentoring Accountability to next level of the system via field reports
Involve a cross-section of stakeholders	Cross-sectional team for content development and planning Multiple levels of Ghana Expanded Programme on Immunization for implementation
Decentralize responsibilities within the cascade structure	Responsibilities diffused as TOT was rolled out; ultimate responsibility was at the district level Periodic and on-demand support from national and regional Expanded Programme on Immunization experts Distance-based support by adult learning expert

Abbreviation: TOT, training of trainers.

### Monitoring and Evaluation Methods

Seven methods were used to monitor and evaluate the intervention, either during the workshop, at the end of the workshop, or after the workshop. The evaluation methods were intended to capture if and how the TOT affected the participants, as well as if and how the trainers applied what they learned. Funding was provided to allow master trainers to conduct mentoring visits. In addition, the project budgeted for an impact evaluation that would be conducted after the end of the entire 2YL initiative. Data were collected at 3 key points: at the end of the TOT workshop, via field activity reports submitted by the trainers for the duration of their training activities, and via surveys of the HCWs after they were trained by DHMTs. Table 3 summarizes the intervention’s monitoring and evaluation methods and the objective(s) that each addressed. A more detailed description follows.

**TABLE 3.** Summary of Ghana Second Year of Life Immunization Training of Trainers Monitoring and Evaluation Methods

**Intervention Phase**	**Method**	**Description**	**Training Objective**
During the workshop	Technical exercises	Exercises using local data to practice new skills and evaluate learning. Facilitators and peers provided feedback.	2YL vaccination services competency
	End of day temperature check	Informal focus group to understand participants’ concerns and adjust for the following day	2YL vaccination services competency
	Teachback feedback	To evaluate learning and give trainers constructive feedback about their training delivery skills	Capacity as a trainer
End of workshop	End of workshop data collection	Activities to collect opinions on training effectiveness and usefulness	2YL vaccination services competency Capacity as a trainer
After workshop, field activities	Activity reporting tool	Electronic survey measuring frequency and process indicators (where, what, who, when) by recording activities that happened at the subdistrict level	Capacity as a trainer
	Field activity reports	Reports submitted from DHMTs following their workforce development intervention at the subdistrict level	Capacity as a trainer
After workshop, evaluation of impact	Health care worker assessment of DHMT trainers (part of the postsurvey)	Survey investigating the value added from the TOT: How did health facility workers perceive the quality of the training they received?	Capacity as a trainer

Abbreviations: 2YL, second year of life; DHMT, district health management team; TOT, training of trainer.

#### During the Workshop

Technical exercises and the teachback were used to evaluate participants’ learning. A daily “temperature check” at day’s end consisted of plenary discussions to get real-time feedback about how each day went and obtain suggestions for the subsequent day. The master trainers conducted daily debriefings to incorporate the suggestions.

#### End of Workshop

The purpose of the end of workshop evaluations was to capture lesson usefulness and workshop quality. We used Thalheimer’s^16^ method of performance-focused surveys to evaluate workshop quality because it aims to avoid positive bias and measurements that are statistically invalid. This method has participants respond to a series of statements and interprets the quality of training based on a rubric developed by the design team that ranks the acceptability of each statement. Table 4 provides a sample question using the Thalheimer method and includes the acceptability rubric that the team defined. Among other reactions, the survey reported participants’ confidence after the workshop from 2 perspectives: their ability to apply what they learned in their workplace and their ability to teach others.

**TABLE 4.** Sample Question From the Training of Trainers Immunization Workshop Evaluation in Ghana

In regard to the technical topics taught, select the SINGLE answer that best describes what the workshop enabled you to do, if anything.	Ranking Standard
It DID NOT enable me to UNDERSTAND NEW CONCEPTS or USE NEW SKILLS.	Unacceptable
It enabled me to UNDERSTAND SOME NEW CONCEPTS but did NOT PREPARE ME TO USE NEW SKILLS on the job.	Unacceptable
It enabled me to BEGIN TRYING NEW SKILLS on the job.	Acceptable
It enabled me to CONFIDENTLY USE NEW SKILLS on the job.	Superior
It enabled me to BE THOROUGHLY CONFIDENT AND PRACTICED IN USING NEW SKILLS on the job.	Superior/ Unlikely

#### After the Workshop: Field Reporting

After each 2YL training activity, DHMTs used *Open Data Kit (ODK)*^17^ (https://getodk.org) software on tablets to report quantitative and qualitative data such as type of training (e.g., workshops, on-the-job training) and when and where trainings occurred. Content was uploaded to a secure cloud server using a mobile data collection platform. They also included their 2YL training activities in the field reports that they were already providing regularly. These written reports enabled the trainers to reflect on their experiences while providing accountability to the higher levels.

#### After the Workshop: Evaluation of Impact as a Trainer

*Impact* for a TOT is defined as the ability of the new trainers to effectively deliver knowledge and skills. Pre- and post-competency assessments were conducted at the health facility level among a systematic random sample of HCWs. As part of the post-assessments, HCWs responded to 7 statements about the quality of the training they received. To define “quality training,” the HCWs were asked questions related to trainers’ subject matter expertise and trustworthiness, the 2 factors that define a “good” trainer.^16^ We present the HCW assessment on the quality of the training, while data on change in HCW competencies are presented by Tchoualeu et al.^18^

## RESULTS

### TOT Participant Characteristics

From July to September 2017, 3 TOT workshops were held: Greater Accra (24 participants), Northern Region (28 participants), and Volta Region (22 participants). Participants included 4 or 5 health managers from each DHMT, the RHMT, and 2 DHMT members from the next planned TOT location (e.g., 2 district managers from Volta attended the Accra workshop and thus took an active role in the subsequent Volta workshop).

### During the Workshop

Participants worked as district teams throughout the workshop, with multiple opportunities for small-group work as well as plenary discussions. Pairs of participants selected their teachback topic and were coached by the master trainers as they prepared their lessons. In addition to suggestions documented on the teachback feedback sheets, teachback debriefs enabled participants to discuss the training techniques they observed from their peers and share how they would apply them in their own trainings. Participants reported appreciating obtaining specific suggestions from the master trainers as well as from their peers.

### End of Workshop

Of the 74 workshop participants, 68 (92%) completed the workshop evaluation survey, responding to 4 questions. Table 5 summarizes the survey responses and shows that participants’ confidence ranged from “able to begin” applying what they learned to “fully confident” in applying both the technical and the training content. Of these 68, 67 (99%) reported a level of confidence that was deemed acceptable or better by the rubric. Fifty-six of 68 responses (82%) related to learning engagement fell into the acceptable range. For the question related to opportunities for practice, participants were invited to agree with multiple statements, 80% of which fell into the acceptable range. Ten responses were unacceptable, and the statement, “I was given too much practice,” defined in the rubric as a red flag, meaning it would require further investigation, received 11 (16%) responses. For the unacceptable and red flag responses, further investigation was done by inspecting the comments sections of the surveys and by interviewing the workshop facilitators. Based on the feedback, the team improved the materials and/or the workshop schedule.

**TABLE 5.** Summary of Ghana Expanded Programme on Immunization Training of Trainers Workshop Evaluation (n=68)

**Survey Statement**		**Rubric Ranking Standard**	**Percentage of Total Responses, All Regions**
Technical content confidence	It did not enable me to understand new concepts or use new skills.	Unacceptable	1
	It enabled me to begin trying new skills on the job.	Acceptable	10
	It enabled me to confidently use new skills on the job.	Superior	35
	It enabled me to be thoroughly confident and practiced in using new skills on the job.	Superior/Unlikely	68
Teaching/mentoring confidence	I am generally comfortable with the concepts taught, but I will need more training/practice to be able to teach or mentor others.	Acceptable	6
	I am able to teach others, but I’ll need more hands-on experience to be fully competent as an instructor.	Acceptable	25
	I am able to teach or mentor others at a fully competent level about the concepts taught.	Superior/Unlikely	66
	I am able to teach or mentor others at an expert level about the concepts taught.	Superior/Unlikely	6
Engaged in the learning	I felt completely uninterested.	Unacceptable	4
	I was often uninterested.	Unacceptable	3
	I was often interested, but sometimes not.	Unacceptable	10
	I was almost always interested.	Acceptable	82
Opportunities to practice	I was given almost no practice.	Unacceptable	1
	I was given inadequate amounts of practice.	Unacceptable	12
	I was given too much practice.	Red Flag	16
	I was often asked to practice something right after we learned it.	Acceptable	53
	I did not get enough helpful feedback when we were practicing.	Unacceptable	1
	I generally received sufficient and helpful feedback after we practiced a task.	Acceptable	71

### After the Workshop: Field Activities

Using the reporting tool, field activities were reported between November 2017 and June 2018. All 5 districts in Greater Accra and 3 of 5 districts each in Northern and Volta Regions submitted reports, reporting a total of 112 activities across the 3 regions.^18^ Most reported that they had conducted workshops (n=65), followed by health facility visits (n=43). Very few reported that they had conducted review meetings (n=4). The TOT participants reported training a total of 1,378 HCWs (Greater Accra=440, Volta=405, Northern=533). Northern Region submitted the greatest number of activity reports (n=60), followed by Volta Region (n=30) and Greater Accra (n=22). DHMTs also reported the topics they taught. The Figure provides a summary of all training activities by region.

**FIGURE:**
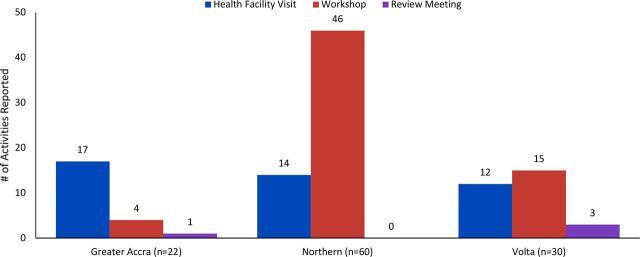
Summary of 112 Reported Training Activities in Greater Accra, Northern, and Volta Regions by Region and Method, in Ghana 2017

### After the Workshop: Evaluation of Impact as a Trainer

In addition to being assessed for knowledge, skill, and attitude, 575 HCWs were surveyed about the training usefulness and facilitator presentation skills: 90 in Greater Accra (Adenta district), 54 in Northern (Tamale Metro), and 431 in Volta (all 5 districts).^17^ Related to increased aptitude, 54% of all respondents reported confidence in using the new skills, with 45% reporting the ability to *begin to try* the new skills. Moreover, 92% of all respondents reported that they would make it a high priority to use their new skills, and 81% reported that the concepts will “help me significantly to improve my work outcomes.” As defined by the analysis rubric agreed upon by the stakeholders, only 1 question received any unacceptable response: 2% of respondents reported that the content would “Will help me *slightly* to improve my work outcomes.” Regarding their experience with the instructors, a question that allowed for multiple responses, all responses fell within the rubric’s definition of acceptable. Of the 575 responses, 300 (52%) reported the instructor “demonstrated a high level of real-world experience,” 162 (28%) reported the instructor “showed deep subject matter expertise,” 377 (66%) reported “I was often asked to practice something right after we learned it,” and 237 (41%) reported, “I generally received sufficient and helpful feedback after we practiced a task.” No unacceptable responses (e.g., “I was given inadequate amounts of practice”) were reported.

## DISCUSSION

The 2YL baseline health facility survey results enabled us to design the TOT intervention based on representative data, not on the speculation of stakeholders.^3^ This information allowed us to address specific 2YL knowledge and practice gaps, such as communicating with caregivers. Integrating operational skill building in the context of completing technical tasks, rather than developing a separate “management” curriculum, recognized that staff could require both types of skills to complete a single task, such as improving defaulter tracking. Devoting half of the class time to practical components of adult learning theory, enabling them to practice-teach in a supportive environment, and setting clear expectations of the new trainers helped prepare the participants in a very practical way. Hayes’ success factors^11^ were a useful check during the training development process.

The 2YL baseline health facility survey results enabled us to design the TOT intervention based on real-world representative data.

Most TOT participants reported increased confidence in applying and teaching the technical content, yet this finding is tempered by studies that have shown workers regularly overestimate their ability.^19^ This phenomenon could explain the notable percentage of responses that our rubric deemed superior/unlikely. Because of the complex nature of the content and our inclusion of mentorship as a component of the intervention, the TOT rubric defined as acceptable if the participants felt less than fully confident at the end of the workshop. Regarding learning engagement, it is important to note that while most participants’ responses were categorized as acceptable by our rubric, the small proportion who reported that they were “often interested but sometimes not” were deemed unacceptable. It may be reasonable to expect adult learners to experience occasional disengagement, and thus care should be taken by training designers when defining their rubrics. We leave it to the reader to determine if it is reasonable to expect that learners will occasionally “tune out.” Finally, we were concerned with the number of unacceptable and red flag responses related to opportunities to practice. These responses were discussed with regional and national leaders in Ghana. The leaders were pleased with the interactivity and requested no major changes to the workshop design.

Regarding the evaluation methods, we found Thalheimer’s^10^ performance-focused survey method more actionable than traditional Likert scale workshop satisfaction surveys, as it not only reduces ambiguity for the participant, but it forces the training designer and stakeholders to think more specifically about the performance improvement they expect from the training. The “most useful lesson” group activity was helpful: in addition to providing the master trainers with timely and detailed feedback, the activity provided an interactive means for participants to review the workshop lessons and envision themselves applying what they learned.

Regarding the field activities, although the trainers chose workshops as their primary training method, it was encouraging to see that it was possible for them to teach the 2YL content using small-group meetings at the health facility or one-on-one coaching, both of which occur on the job with minimal disruption to the workflow. The master trainers who observed the DHMT-led workshops noticed a high degree of comfort with the technical content as well as the application of the adult learning principles that were stressed in the workshops. Through the field activity reports, we were able to partially track the follow-through that Hahn et al.^8^ and Orfaly et al.^9^ recommended.

Based on the participants’ self-reported technical and training confidence, their action plans, and their field reports, the stakeholders considered the TOT a success. The cross-functional team, composed of technical EPI and workforce development experts, supported an intervention that was technically accurate and practical to deploy in Ghana’s setting. Yet none of these factors were as influential as the posttraining actions in the 3 regions: the regional and district teams were open to taking the time to learn new ways of doing their own work and training others in a purposeful way.

Based on the participants’ self-reported technical and training confidence, their action plans, and their field reports, the TOT was considered a success.

We appreciate that all these practices may not be possible in low-resource settings. Still, Pearce et al.^5^ suggested that a combination of teaching techniques can help to effectively disseminate content to health workers. We propose, then, that the following combination of factors can support an effective TOT:

- Use learning science to inform analysis, design, delivery, and evaluation of the intervention.
- Define specific performance gaps and expected behavior change to inform the content and learning activities.
- Use a variety of components such as those described in Table 2 to build capacity as trainers.
- Spend enough class time to prepare participants for their training role.
- Ensure that the technical content is practical (i.e., activity based) and not just a review of theories or policies.
- Provide instructor materials such as lesson outlines that sketch out how to conduct the lesson.
- Use performance-focused evaluation methods such as Thalheimer’s^10^ to add rigor to the workshop evaluations.
- Give the trainers specific tasks and expectations, then monitor their field activities.
- Budget for trainer field support and postintervention evaluation.
- Engage regional and local support for the trainers.

### Limitations

With the availability of technology and acceptance of text messaging platforms in even the most remote areas, we think that we could have integrated technology into the design to a greater extent: peer networks and electronic checklists could provide valuable performance support.

Since field activities were reported at the district level without direct oversight of the project team, we were unable to calculate the number of TOT participants who went on to teach. The lack of resources for a field monitoring team also prevented us from conducting a more rigorous evaluation of the participants’ impact as a trainer, and we were only able to rely on the learners’ perceptions. Additionally, the complete absence of reporting for 2 of the 5 districts in both Northern and Volta regions demonstrates that we cannot know how many activities did occur that were simply not reported. In future efforts, we will engage the trainers earlier in the project to determine a more realistic means for collecting data. We are concerned about sustainability and are looking forward to learning if and how TOT practices continue if external funding and technical support are absent. We also await the findings of the end-of-project evaluation to see if and how performance at the health facility level has improved.

## CONCLUSIONS

Our experience demonstrates how the best practices of adult learning and TOT can support performance-focused training that is applied on the job, although the specifics of the Ghana 2YL initiative should be adapted for local circumstances. We hope the description of methods and tools is specific enough that readers can adapt them for their context. We also believe our findings reinforce the importance of conducting a performance-based baseline assessment and for budgeting resources to enable rigorous monitoring and evaluation. Finally, regardless of the circumstances, strong collaboration and a united purpose such as that demonstrated by the Ghanaian EPI staff at the national, regional, district, and health facility levels created an environment that supported the 2YL TOT.

## Supplementary Material

21-00090-Traicoff-Supplement.pdf

## Notes

**Peer Reviewed**
