# Supplementary material for: Applying Adult Learning Best Practices to Design Immunization Training for Health Care Workers in Ghana
Source: Glob Health Sci Pract. 2021 Sep 30;9(3):487–97. doi: 10.9745/GHSP-D-21-00090 (PMC8514033; doi:10.9745/GHSP-D-21-00090)
Supplement: 21-00090-Traicoff-Supplement.pdf [file 21-00090-Traicoff-Supplement.pdf]

Ghana 2YL TOT Workshop
Detailed Session Plan: Training the Trainers

Session Objectives

It is expected that after these sessions, participants will be able to:

- Develop a customized training plan
- Demonstrate the ability to teach a lesson for 2YL
- Complete the competency tool, either using paper based and mobile app

Learning Objectives

- Describe the characteristics of adult learners
- Use techniques in the classroom to encourage interactivity and learning
- Prepare a personal development plan to improve as an instructor
- Describe the roles and responsibilities for implementing 2YL training
- Select strategies for changing training activity type for specific training needs
- Complete a baseline assessment using the competency tool

Materials Needed
Topic signup sheet<br>Trainer instructions<br>Individual development plans<br>Challenging participants handout<br>Participants version of 2YL lesson plan<br>Classroom supplies<br>Trainer feedback forms

Key Prerequisite Knowledge
Technical knowledge

Lesson	Key Points/Content	Day/Time	Instructional Activity/Source Materials
Introduction	Expectations of workshop	Day 1/5 min	As part of Welcome during Session 1<br>Post TOT Signup sheet
Training Adults	Intro to adult learning	20 min	ppt
Exercise	Exercise 1: Dos & Don'ts	30 min	1. Flip chart<br>2. Sticky notes
Daily Review	Review day from trainer's point of view: point out key lessons, trainer tips	15 min	Show example of session outlines
Training Adults	Training delivery good practices	Day 2/30 min	ppt
Exercise	Exercise 2: Manage the classroom	25 min	1. Handling challenging situations handout<br>2. Scenarios

Lesson	Key Points/Content	Day/Time	Instructional Activity/Source Materials
	Next steps	5 min	
Sign up for Teachback	Form pairs & choose a topic from the list	5 min	Sign up during lunch on Day 2<br>Distribute preparation instructions
Daily Review	Review day from trainer's point of view: point out key lessons, trainer tips	15 min	
Planning training activities	Role of trainers<br>5 moments of need	Day 3/20 min	Ppt/discussion
Exercise	Exercise 1: Assess the level of risk	10 min	
Selecting training activities	Learning strategies	15 min	Ppt/discussion
Daily Review	Review day from trainer's point of view: point out key lessons, trainer tips<br>Include guidance for facilitating a Process Improvement workshop	15 min	
Developing training plans	Workshop: develop preliminary plans<br>Present plans	Day 4/2.25 hrs	
M&E tools	Explain M&E tools & process	45 min	Demonstration/discussion
Teachback	Prepare teachback sessions	1 hr	
Teachback	Begin: Teach sample lessons	1 hr	1. Teams of 2 teach for maximum 20 minutes, with other participants acting as 'students'<br>2. Faculty completes feedback forms<br>3. Other participants provide feedback using informal method (to be determined)
Daily Review	Review day from trainer's point of view: point out key lessons, trainer tips	15 min	

Lesson	Key Points/Content	Day/Time	Instructional Activity/Source Materials
	Include guidance for facilitating a Process Improvement workshop		
Teachback	Complete: Teach sample lessons	Day 5/4.5 hrs	4. Teams of 2 teach for maximum 20 minutes, with other participants acting as 'students'<br>5. Faculty completes feedback forms<br>6. Other participants provide feedback using informal method (to be determined)
Teachback	Teachback debrief	30 min	Discuss the experience & next steps
**Total Time**		**13.75 hrs**	

Ghana 2YL TOT
Teachback Instructions

To help you prepare for the teachback session, please review this instruction sheet.

1. By now you should have met with your teammate and selected a topic from the list.
2. Decide which teammate will be lead instructor for this session. The other teammate will be the co-trainer. You may divide responsibilities as you wish.
3. Prepare a short (15 minutes) lesson on the topic using the following guidelines:
   a. Use the Ghana 2YL materials to help you prepare your lesson.
   b. Be sure to include instruction of content and an exercise to support the content.
   c. If you wish, you may also include a ‘workshop’ introduction, icebreaker, energizer or assessment. Any of these activities must fit in the 15 minute time frame.
   d. Use the principles of adult learning, course materials, textbooks, and any materials you brought with you.
   e. Prepare handouts as possible.

Tips for a positive teachback experience

1. RELAX!! No one expects you to be an expert at the subject matter. Remember the purpose of this activity is to allow you to practice your new skills in a friendly environment where you can receive constructive feedback.
2. As others train, you will be a ‘student’ in their class. This is excellent time to remember the Golden Rule (“Do unto others as you would have them do unto you”). Feel free to role play a ‘typical’ student, but remain positive and supportive of your classmate.
3. You will have the opportunity to provide feedback to each other.

Teachback Feedback Form
Ghana 2YL

Topic :____

Co-Instructor:____________________**Co-Instructor:**______________________

		Check One Excellent	Good	Fair	Constructive Comments
**Subject Matter**	Showed understanding of content				
	Was able to answer questions				
	Included key points				
**Adult Learning**	Used multiple delivery methods				
	Encouraged participation				
	Used exercise to support learning				
**Delivery**	Used clear learning objectives				
	Began with a strong opening				
	Managed time well				
	Appeared confident				
**Materials**	Organized lesson in logical order				
	Used visual aids effectively				

Comments:

I liked:

____

____

I wish:

____

____

Use the other side of this form for additional comments

GHANA 2YL CONFIDENT INSTRUCTOR
Personal Development Plan

My Most Important Do's and Don'ts

Are you worried about:	Then try:	Check the items you want to work on
Fear	Be well prepared	
	Use ice breakers	
	Acknowledge the fear (to yourself!)	
Credibility	Don't apologize	
	Have an attitude of an expert	
	Share personal background	
Personal Experiences	Report personal experiences	
	Report experiences of others	
	Use analogies, movies or famous people	
Difficult Learners	Confront problem learner	
	Circumvent dominating behavior	
	Use small groups for timid behavior	
Participation	Ask open-ended questions	
	Plan small group activities	
	Invite participation	
Timing	Plan well	
	Practice, practice, practice	
	Allow time for questions	
Adjust Instruction	Know group needs	
	Request feedback	
	Redesign during breaks	
Questions	Anticipate questions	
	Paraphrase learners' questions	
	Admit if you don't know	
Feedback	Solicit informal feedback	
	Evaluate the class	
	Observe other instructors	
Media, Materials, Facilities	Know the equipment	
	Have materials ready	
	Have a back-up plan	
Openings & Closings	Develop an 'openings' file	
	Relax the students	
	Summarize concisely	
Dependence on Notes	Use cards	
	Use visuals	
	Practice	

Ghana 2YL Workshop
Conclusion Session & Follow Up

**Materials Needed**
Flip Chart<br>Markers<br>Health worker workshop evaluation survey

**Outline**	**Content**	**Time**	**Instructional Activity**
Workshop Review	Summarize the workshop	10 minutes	Instruct teams to work in their groups to develop their “Top 10” list: List the 10 lessons that they found most useful.
	Activity Debrief	10 minutes	Have teams report their Top 10 list. Lead brief discussions as appropriate.
Workshop Survey			1. Distribute 1 page survey. Instruct participants to use the back of the survey to add comments or suggestions for improvement.<br>2. While participants are completing the survey, take photos of the flip charts or record the teams’ Top 10 lists.<br>3. Collect the evaluation forms.
Closing Remarks		5 minutes	Thank the participants for their participation.<br>Remind them of available resources.
**Total Time**		**30 minutes**	

**POST WORKSHOP**	1. Summarize the evaluation survey responses within 1 week of the training. Include all comments.<br>2. Use the taxonomy below to assess the success of the workshop.<br>3. Summarize the Top 10 lists to develop a list of most useful lessons.<br>4. Submit the evaluation summary & most useful lessons to XXXX within 1 month of the training.

Ghana 2YL Health Worker and Supervisor Workshop

Suggested Ranking Standards

1. Check the **one best answer** that best describes what the workshop enabled you to do, if anything.		
	A. It DID NOT enable me to UNDERSTAND NEW CONCEPTS or USE NEW SKILLS.	Unacceptable
	B. It enabled me to UNDERSTAND SOME NEW CONCEPTS, but did NOT PREPARE ME TO USE NEW SKILLS on the job.	Unacceptable
	C. It enabled me to BEGIN TRYING NEW SKILLS on the job.	Acceptable
	D. It enabled me to CONFIDENTLY USE NEW SKILLS on the job.	Superior
	E. It enabled me to BE THOROUGHLY CONFIDENT AND PRACTICED IN USING NEW SKILLS on the job.	Superior/ Unlikely
2. In regard to the concepts taught in the workshop, how motivated WILL YOU BE to USE these skills in your work? **Check the one best answer**.		
	A. I WILL NOT MAKE THIS A PRIORITY when I get back to my job.	Unacceptable
	B. I will make this a PRIORITY-BUT A LOW PRIORITY-when I get back to my job.	Unacceptable
	C. I will make this a MODERATE PRIORITY when I get back to my job.	Acceptable
	D. I will make this a HIGH PRIORITY when I get back to my job.	Acceptable
	E. I will make this one of my HIGHEST PRIORITIES when I get back to my job	Superior
3. Which of the following were true about the opportunities you were given to practice? **Check all that apply**.		
	A. I was given ALMOST NO PRACTICE.	Unacceptable
	B. I was given INADEQUATE AMOUNTS OF PRACTICE.	Unacceptable
	C. I was given TOO MUCH PRACTICE.	Red Flag
	D. I DID NOT GET ENOUGH HELPFUL FEEDBACK WHEN WE WERE PRACTICING.	Unacceptable
	E. I was OFTEN ASKED TO PRACTICE SOMETHING RIGHT AFTER WE LEARNED IT.	Acceptable
	F. I generally RECEIVED SUFFICIENT AND HELPFUL FEEDBACK AFTER WE PRACTICED A TASK.	Acceptable
4. Which of the following were true about your instructor? **Check all that apply.**		
	A. Was OFTEN UNCLEAR or DISORGANIZED	Unacceptable
	B. Exhibited UNACCEPTABLE LACK OF KNOWLEDGE.	Unacceptable
	C. Exhibited LACK OF REAL-WORLD EXPERIENCE.	Unacceptable
	D. Generally PERFORMED COMPETENTLY AS A TRAINER.	Acceptable
	E. Showed DEEP SUBJECT-MATTER KNOWLEDGE.	Superior
	F. Demonstrated HIGH LEVELS OF REAL-WORLD EXPERIENCE.	Acceptable
	G. MOTIVATED ME TO ENGAGE ACTIVELY IN THE LEARNING.	Acceptable
	H. Is a person I CAME TO TRUST.	Superior
5. From your perspective, how valuable are the concepts taught in the workshop? HOW MUCH WILL THEY HELP YOU IMPROVE YOUR MEASLES-RUBELLA/MEN A COVERAGE OUTCOMES?		
	A. Will NOT HELP ME to improve my outcomes.	Unacceptable
	B. Will HELP ME SLIGHTLY to improve my work outcomes.	Unacceptable
	C. Will HELP ME TO A MODERATE AMOUNT to improve my work outcomes.	Acceptable
	D. Will HELP ME SIGNIFICANTLY to improve my work outcomes.	Superior
	E. Concepts taught ARE NOT RELEVANT to my work.	Red Flag

Sample Evaluation Summary Report

The following pages display an example of a summary report. You can use this as your template. Be sure to include the number of participants (N=X)

Evaluation Report Template for X location. Workshop Date:

II End of workshop activity: Most useful lessons

Working in their teams, participants were asked to list their top 10 lessons, based on expected usefulness. There were 5 groups.

Detailed results are listed below:

**Lesson/Key Word**	**Number of times mentioned**
List responses here	

II End of workshop survey summary

N=*XX*

			Responses
1. In regard to the topics taught, select the answer that best describes what the workshop enabled you to do, if anything.			
	A. It DID NOT enable me to UNDERSTAND NEW CONCEPTS or USE NEW SKILLS.	Unacceptable	
	B. It enabled me to UNDERSTAND SOME NEW CONCEPTS, but did NOT PREPARE ME TO USE NEW SKILLS on the job.	Unacceptable	
	C. It enabled me to BEGIN TRYING NEW SKILLS on the job.	Acceptable	
	D. It enabled me to CONFIDENTLY USE NEW SKILLS on the job.	Superior	
	E. It enabled me to BE THOROUGHLY CONFIDENT AND PRACTICED IN USING NEW SKILLS on the job.	Superior/ Unlikely result	
2. In regard to the concepts taught in the workshop, how motivated WILL YOU BE to USE these skills in your work?			
	A. I WILL NOT MAKE THIS A PRIORITY when I get back to my job.	Unacceptable	
	B. I will make this a PRIORITY-BUT A LOW PRIORITY-when I get back to my job.	Unacceptable	
	C. I will make this a MODERATE PRIORITY when I get back to my job.	Acceptable	
	D. I will make this a HIGH PRIORITY when I get back to my job.	Acceptable	
	E. I will make this one of my HIGHEST PRIORITIES when I get back to my job	Superior	
3. Which of the following were true about the opportunities you were given to practice?			
	A. I was given ALMOST NO PRACTICE.	Unacceptable	
	B. I was given INADEQUATE AMOUNTS OF PRACTICE.	Unacceptable	
	C. I was given TOO MUCH PRACTICE.	Red Flag	
	D. I DID NOT GET ENOUGH HELPFUL FEEDBACK WHEN WE WERE PRACTICING.	Unacceptable	
	E. I was OFTEN ASKED TO PRACTICE SOMETHING RIGHT AFTER WE LEARNED IT.	Acceptable	
	F. I generally RECEIVED SUFFICIENT AND HELPFUL FEEDBACK AFTER WE PRACTICED A TASK.	Acceptable	
4. Which of the following were true about your instructor?			
	A. Was OFTEN UNCLEAR or DISORGANIZED	Unacceptable	
	B. Exhibited UNACCEPTABLE LACK OF KNOWLEDGE.	Unacceptable	
	C. Exhibited LACK OF REAL-WORLD EXPERIENCE.	Unacceptable	
	D. Generally PERFORMED COMPETENTLY AS A TRAINER.	Acceptable	
	E. Showed DEEP SUBJECT-MATTER KNOWLEDGE.	Superior	
	F. Demonstrated HIGH LEVELS OF REAL-WORLD EXPERIENCE.	Acceptable	
	G. MOTIVATED ME TO ENGAGE ACTIVELY IN THE LEARNING.	Acceptable	
	H. Is a person I CAME TO TRUST.	Superior	
5. From your perspective, how valuable are the concepts taught in the workshop? HOW MUCH WILL THEY HELP YOU IMPROVE YOUR MEASLES COVERAGE OUTCOMES?			
	A. Will NOT HELP ME to improve my outcomes.	Unacceptable	
	B. Will HELP ME SLIGHTLY to improve my work outcomes.	Unacceptable	
	C. Will HELP ME TO A MODERATE AMOUNT to improve my work outcomes.	Acceptable	
	D. Will HELP ME SIGNIFICANTLY to improve my work outcomes.	Superior	
	E. Concepts taught ARE NOT RELEVANT to my work.	Red Flag	

Summary of comments

Is there one specific thing you plan to do differently when you return to your workplace?

List responses here
